# Association between dietary knowledge and overweight and obesity in Chinese children and adolescents: Evidence from the China Health and Nutrition Survey in 2004–2015

**DOI:** 10.1371/journal.pone.0278945

**Published:** 2022-12-09

**Authors:** Zhen Xu, Yibin Zhao, Jingjing Sun, Lisi Luo, Yu Ling

**Affiliations:** 1 Department of Child healthcare, Kunming Children’s Hospital, Kunming, Yunnan, China; 2 Department of Child Rehabilitation, Kunming Children’s Hospital, Kunming, Yunnan, China; Zagazig University Faculty of Human Medicine, EGYPT

## Abstract

**Objective:**

To assess whether dietary knowledge of Chinese children and adolescents and their mothers was associated with childhood and adolescent overweight and obesity.

**Methods:**

This cross-sectional study obtained data from the China Health and Nutrition Survey (CHNS) between 2004 and 2015. Dietary knowledge of children and adolescents and their mothers was assessed by asking questions and statements on diets, and clustered by K-means clustering. Body mass index (BMI) and waist circumference (WC) were used to evaluate overweight and obesity among children and adolescents. The association of dietary knowledge with childhood and adolescent overweight and obesity was evaluated by multivariate regression analysis, with odds ratios (ORs) and 95% confidence intervals (CIs) calculated.

**Results:**

A total of 2,338 children and adolescents were included. Children and adolescents with low dietary knowledge were demonstrated to have significantly higher risks of BMI-defined overweight or obesity (OR = 1.66, 95%CI = 1.21–2.28, *P* = 0.002), and WC-defined obesity (OR = 1.52, 95%CI = 1.12–2.06, *P* = 0.007) than those with high dietary knowledge. Compared with high dietary knowledge in mothers, low dietary knowledge was associated with significantly elevated risks of BMI-defined overweight or obesity (OR = 1.48, 95%CI = 1.08–2.02, *P* = 0.014), and WC-defined obesity (OR = 1.59, 95%CI = 1.18–2.16, *P* = 0.003). Furthermore, significantly increased odds of BMI-defined overweight or obesity and WC-defined non-obesity in children and adolescents were related to low dietary knowledge versus high dietary knowledge of children and adolescents (OR = 1.72, 95%CI = 1.08–2.74, *P* = 0.023), while there was no association of BMI-defined non-overweight and non-obesity and WC-defined obesity with dietary knowledge among children and adolescents (OR = 1.35, 95%CI = 0.89–2.04, *P* = 0.161). Additionally, no association was found between dietary knowledge of mothers and BMI-defined overweight or obesity and WC-defined non-obesity among children and adolescents (OR = 1.39, 95%CI = 0.89–2.17, *P* = 0.155), while low dietary knowledge of mothers was associated with increased odds of BMI-defined non-overweight and non-obesity and WC-defined obesity in children and adolescents (OR = 1.58, 95%CI = 1.03–2.43, *P* = 0.036).

**Conclusion:**

Dietary knowledge of children and adolescents and their mothers was associated with childhood and adolescent overweight and obesity. Dietary knowledge of children and adolescents negatively related to the risk of BMI-defined overweight or obesity, and dietary knowledge of mothers to odds of WC-defined obesity.

## Introduction

Overweight and obesity in children and adolescents has become one of the most serious public health problems of the 21st century, with significantly increasing worldwide prevalence [1, 2]. In China, the prevalence of overweight and obesity among children and adolescents increased from 5.0% and 1.7% in 1991–1995 to 11.7% and 6.8% in 2011–2015, respectively [3]. In 2017, the prevalence rose to 15.1% and 10.7% for overweight and obesity, separately [4]. Childhood and adolescent obesity tracks adulthood obesity and is associated with many chronic diseases, including type 2 diabetes, hypertension, and cardiovascular disease, and further relates to adulthood mortality and premature death [5]. Hence, identifying overweight and obesity-related factors is important to improve health for children and adolescents.

Despite growing prevalence and unfavorable outcomes, overweight and obesity are multifactorial conditions affected by genetic and non-genetic factors [6]. For children and adolescents, these states were generally resulted from a lack of physical activity, unhealthy dietary patterns leading to excess energy intake, or both [2, 7]. Thereinto, diet intake, variety, and quality play important roles in overweight and obesity of children and adolescents [8–11]. Asakura *et al*. [12] reported that nutrition knowledge was related to dietary intake in primary school children in Japan. Knowledge about diets was found to be associated with overweight and obesity in adults, and eating self-regulation based on dietary knowledge can predict adulthood overweight and obesity [13]. Nutrition knowledge was also identified by Akkartal *et al*. [14] to correlate with diet quality and obesity in adults. Currently, body mass index (BMI) and waist circumference (WC) are the most common indicators used to determine overweight and obesity in children and adolescents, with great diagnostic performances [15]. WC is a good marker of abdominal obesity and BMI is a marker of increases in overall adiposity [16]. Most prior studies assessed overweight and obesity among children and adolescents with BMI [17–19]. Despite the wide use and ease of obtaining BMI, this index has low sensitivity to detect excess adiposity, does not measure actual body fat, and cannot distinguish accurately between fat and fat-free mass [20–22]. Additionally, the association between dietary knowledge and overweight and obesity in children and adolescents defined by BMI and WC remains unexplored.

The aim of this study was to evaluate whether dietary knowledge of Chinese children and adolescents and their mothers was associated with their overweight and obesity defined by BMI and WC, based on the China Health and Nutrition Survey (CHNS).

## Methods

### Study population

This cross-sectional study obtained data on Chinese children and adolescents from the China Health and Nutrition Survey (CHNS) between 2004 and 2015. The CHNS used a multistage random-cluster sampling process to select samples from 15 provinces in China [23]. The CHNS was approved by the Institutional Review Committees of the University of North Carolina at Chapel Hill and the National Institute of Nutrition and Food Safety, Chinese Center for Disease Control and Prevention. Children and adolescents aged 8–18 years were included. Children and adolescents who and whose mothers did not have dietary knowledge levels measured, and who have no data on height, weight and waist circumference were excluded from the study. This study did not need to be approved by the Institutional Review Board of Kunming Children’s Hospital because the data were accessed from the CHNS (a publicly available database). Written informed consent was obtained from all participants.

### Dietary knowledge of children and adolescents and their mothers

Dietary knowledge of children and adolescents were assessed by asking the respondent the following questions and statements:

1. Do you know about the dietary guidelines for Chinese residents? (abbreviated as DG)
2. Choosing a diet with a lot of fresh fruits and vegetables is good for one’s health. (abbreviated as LF)
3. Eating a lot of sugar is not good for one’s health. (abbreviated as VS)
4. Eating a variety of foods is good for one’s health. (abbreviated as VF)
5. Choosing a diet high in fat is not good for one’s health. (abbreviated as HF)
6. Choosing a diet with a lot of staple foods (rice and rice products and wheat and wheat products) is not good for one’s health. (abbreviated as SF)
7. Consuming a lot of animal products daily (fish, poultry, eggs and lean meat) is not good for one’s health. (abbreviated as AP)
8. Reducing the amount of fatty meat and animal fat in the diet is good for one’s health. (abbreviated as AM)
9. Consuming milk and dairy products is good for one’s health. (abbreviated as MDP)
10. Consuming beans and bean products is good for one’s health. (abbreviated as BBP)
11. Physical activities are good for one’s health. (abbreviated as PA)
12. Sweaty sports or other intense physical activities are not good for one’s health. (abbreviated as IPA)
13. The heavier one’s body is, the healthier he or she is. (abbreviated as HB)
14. For mothers: the priority of “having my child be physically active” in your life. (abbreviated as CPA)
15. For mothers: the priority of “having my child eat a healthy diet” in your life. (abbreviated as CEH)

Regarding the item (1), the answer “no” was coded as “0”, and “yes” as “1”. Regarding the items (2)-(13), the response “strongly disagree” was coded as “1”, “disagree” as “2”, “neutral” as “3”, “agree” as “4”, and “strongly agree” as “5”. Regarding the items (14)-(15), the response “not important at all” was coded as “1”, “not very important” as “2”, “important” as “3”, “very important” as “4”, and “the most important” as “5”. Then the dietary knowledge of children and adolescents, and their mothers was clustered by K-means clustering.

### Overweight and obesity in children and adolescents

BMI and WC were used to evaluate overweight and obesity among children and adolescents. International cut-offs of BMI for overweight and obesity by gender between 2 and 18 years of age were shown previously [24]. International 90th percentile WC cut-offs for central obesity in children and adolescent aged 6–18 years were also illustrated before [25].

### Other variables

Data about age (years), gender, systolic blood pressure (SBP, mmHg), diastolic blood pressure (DBP, mmHg), smoking, alcohol use, television time in a week (including weekdays and a weekend) (min), area, province, hip circumference (cm), and mother’s education level were collected from the CHNS. Area included urban and rural arears. Province was classified into eastern provinces (Beijing, Shanghai, Jiangsu, Shandong, Zhejiang), central provinces (Henan, Hubei, Hunan), western provinces (Guizhou, Guangxi, Chongqing, Shaanxi, Yunnan), and northeastern province (Heilongjiang, Liaoning). Mother’s education level was divided into none, graduate from primary school, lower middle school degree, upper middle school degree, technical or vocational degree, and university or college degree or higher.

### Statistical analysis

Normally distributed measurement data were described as mean ± standard deviation (Mean ± SD), and the independent samples t-test was used for comparison between groups; non-normal data were expressed as median and quartile [M (Q_1_, Q_3_)], and the Mann-Whitney U rank sum test was applied for intergroup comparison; enumeration data were shown by the number of cases and constituent ratio [n (%)], and the Chi-square test or Fisher’s exact test was employed for comparison between groups. Missing data were subjected to multiple imputation (R: mice), and sensitivity analysis was performed via difference analysis on the data before and after imputation. The number of clusters was determined, and K-means clustering was used to cluster dietary knowledge of children and adolescents, and their mothers, respectively. The screened confounding factors were put into multivariate models as covariates, along with dietary knowledge of children and adolescents. Multivariate regression analysis was conducted with overweight and obesity defined by BMI and WC as the outcome variables, with odds ratios (ORs) and 95% confidence intervals (CIs) calculated. Model 1 was a univariate model, model 2 was adjusted for age, gender, and mother’s education level. Model 3 was adjusted for age, gender, mother’s education level, geographic location (area and province), SBP, and DBP. In model 3, television time in a week was additionally corrected for when obesity in children and adolescents was evaluated via WC. All statistical tests were two-sided, and *P* < 0.05 was considered statistically significant. Statistical analysis was performed using SAS 9.4 (SAS Institute Inc., Cary, NC) and R 4.20 (R Foundation for Statistical Computing, Vienna, Austria) software.

## Results

### The characteristics of the study population

**Fig 1:**
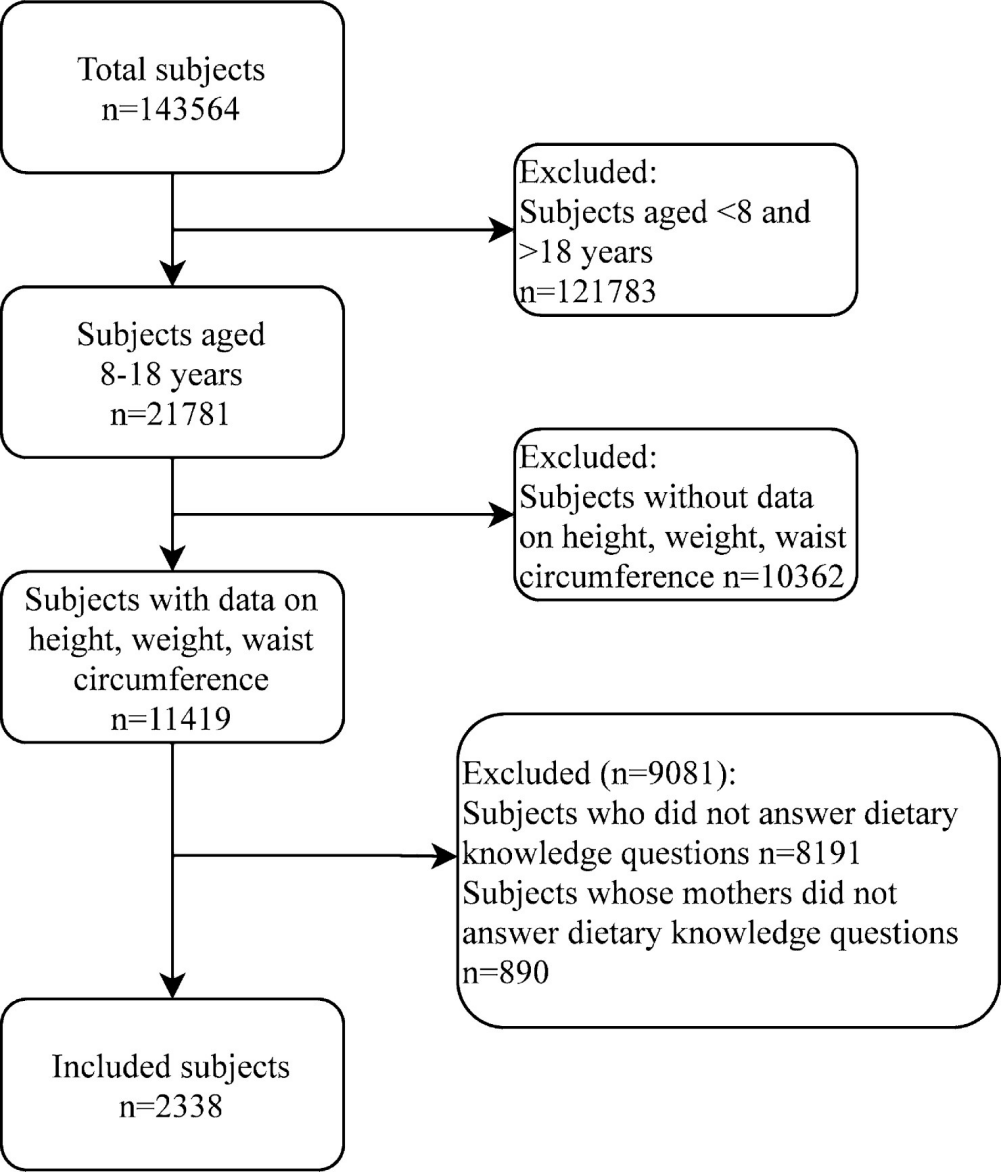
Flow chart of study population selection.

**Fig 2:**
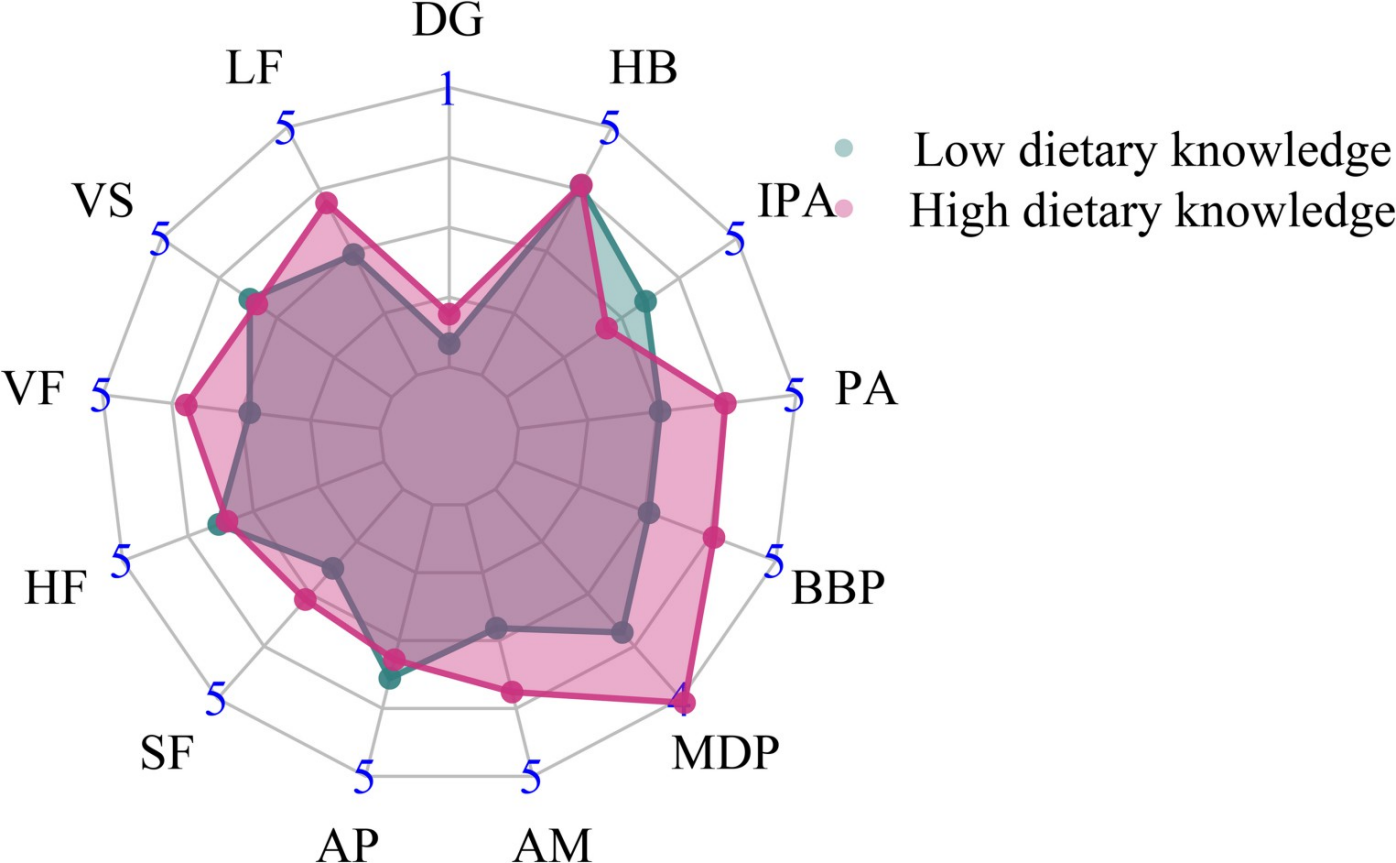
Radar plot for dietary knowledge of children and adolescents. DG: Do you know about the dietary guidelines for Chinese residents? LF: Choosing a diet with a lot of fresh fruits and vegetables is good for one’s health. VS: Eating a lot of sugar is not good for one’s health. VF: Eating a variety of foods is good for one’s health. HF: Choosing a diet high in fat is not good for one’s health. SF: Choosing a diet with a lot of staple foods (rice and rice products and wheat and wheat products) is not good for one’s health. AP: Consuming a lot of animal products daily (fish, poultry, eggs and lean meat) is not good for one’s health. AM: Reducing the amount of fatty meat and animal fat in the diet is good for one’s health. MDP: Consuming milk and dairy products is good for one’s health. BBP: Consuming beans and bean products is good for one’s health. PA: Physical activities are good for one’s health. IPA: Sweaty sports or other intense physical activities are not good for one’s health. HB: The heavier one’s body is, the healthier he or she is.

**Fig 3:**
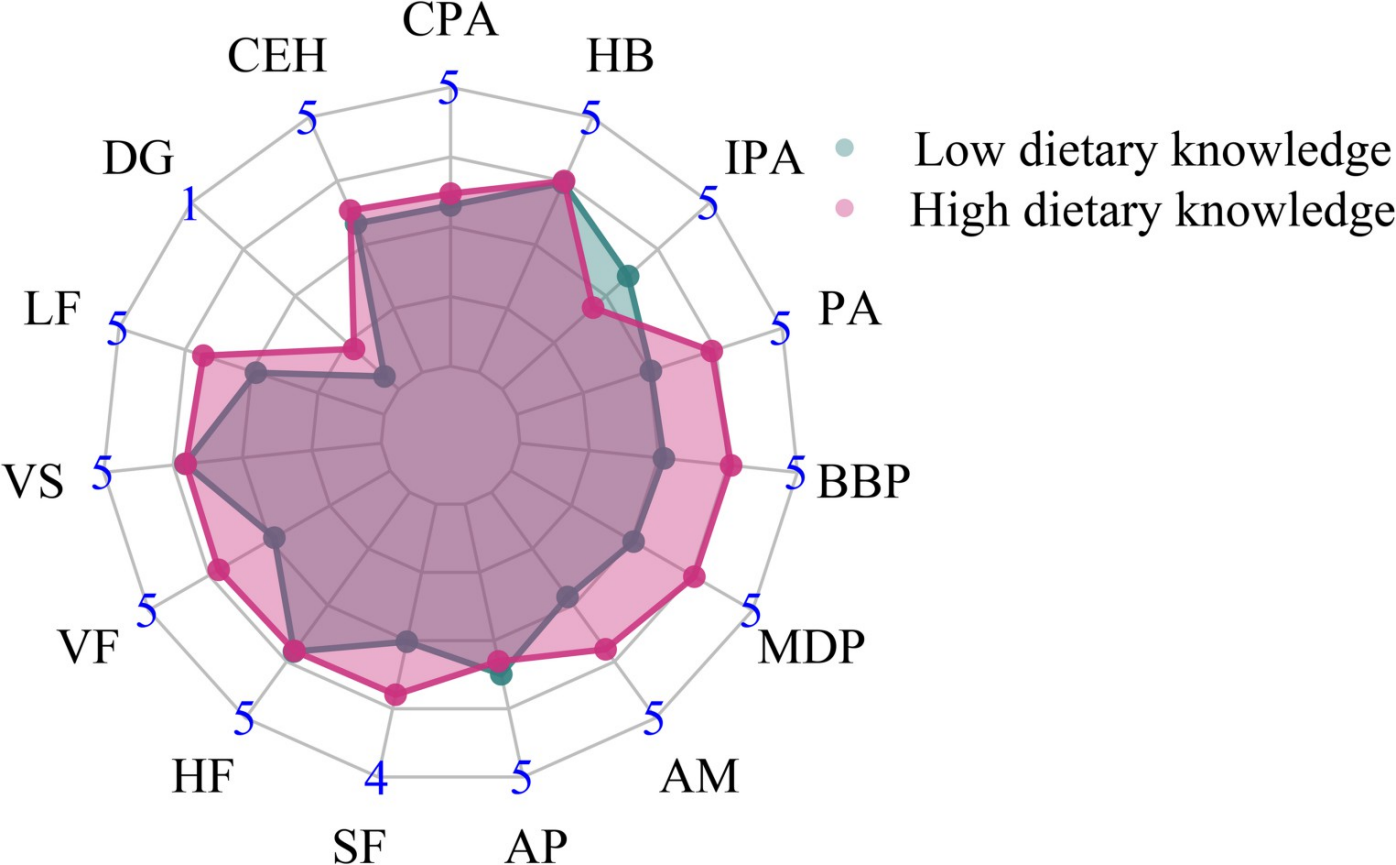
Radar plot for dietary knowledge of mothers. DG: Do you know about the dietary guidelines for Chinese residents? LF: Choosing a diet with a lot of fresh fruits and vegetables is good for one’s health. VS: Eating a lot of sugar is not good for one’s health. VF: Eating a variety of foods is good for one’s health. HF: Choosing a diet high in fat is not good for one’s health. SF: Choosing a diet with a lot of staple foods (rice and rice products and wheat and wheat products) is not good for one’s health. AP: Consuming a lot of animal products daily (fish, poultry, eggs and lean meat) is not good for one’s health. AM: Reducing the amount of fatty meat and animal fat in the diet is good for one’s health. MDP: Consuming milk and dairy products is good for one’s health. BBP: Consuming beans and bean products is good for one’s health. PA: Physical activities are good for one’s health. IPA: Sweaty sports or other intense physical activities are not good for one’s health. HB: The heavier one’s body is, the healthier he or she is. CPA: the priority of “having my child be physically active” in your life. CEH: For mothers: the priority of “having my child eat a healthy diet” in your life.

Of 2,338 included children and adolescents, 1,251 were males and 1,087 were females. The mean age of these children and adolescents was 14.68 years. The majority of the children and adolescents did not smoke (96.45%) or drink (91.87%), and lived in rural areas (66.25%). Most mothers had a lower middle school degree (40.98%). The flow chart of study population selection is shown in Fig 1. Table 1 illustrates the characteristics of the included children and adolescents. According to overweight and obesity defined by BMI, the children and adolescents were divided into normal weight group by BMI, and overweight or obese group by BMI. In term of obesity defined by WC, these children and adolescents were classified into non-obese group by WC and obese group by WC. Age, gender, SBP, DBP, province, hip circumference, and mother’s education level were found to be significantly different between the normal weight group and the overweight or obese group by BMI (all *P* < 0.05). For the non-obese and obese groups by WC, significant differences existed in SBP, DBP, area, province, hip circumference, and mother’s education level (all *P* < 0.05). Besides, two clusters (low dietary knowledge, high dietary knowledge) were established for dietary knowledge of children and adolescents (Fig 2), and two clusters (low dietary knowledge, high dietary knowledge) were also developed for dietary knowledge of mothers, using K-means clustering (Fig 3).

**Table 1:** Characteristics of the included children and adolescents.

Variables	Total (n = 2338)	Normal weight group by BMI (n = 2108)	Overweight or obese group by BMI (n = 230)	Statistics	*P*	Non-obese group by WC (n = 2093)	Obese group by WC (n = 245)	Statistics	*P*
Age (years), Mean±SD	14.68 ± 1.99	14.73 ± 1.99	14.20 ± 2.00	t = 3.79	<0.001	14.70 ± 1.99	14.45 ± 1.99	t = 1.85	0.064
Gender, n(%)				χ^2^ = 12.051	<0.001			χ^2^ = 0.155	0.694
Male	1251 (53.51)	1103 (52.32)	148 (64.35)			1117 (53.37)	134 (54.69)		
Female	1087 (46.49)	1005 (47.68)	82 (35.65)			976 (46.63)	111 (45.31)		
SBP (mmHg), Mean±SD	105.99 ± 11.46	105.43 ± 11.19	111.12 ± 12.63	t = -6.55	<0.001	105.38 ± 11.20	111.18 ± 12.33	t = -7.02	<0.001
DBP (mmHg), Mean±SD	69.65 ± 8.55	69.32 ± 8.41	72.74 ± 9.22	t = -5.80	<0.001	69.18 ± 8.40	73.68 ± 8.75	t = -7.89	<0.001
Smoking, n(%)				χ^2^ = 2.071	0.150			χ^2^ = 0.065	0.799
No	2255 (96.45)	2037 (96.63)	218 (94.78)			2018 (96.42)	237 (96.73)		
Yes	83 (3.55)	71 (3.37)	12 (5.22)			75 (3.58)	8 (3.27)		
Alcohol use, n(%)				χ^2^ = 0.468	0.494			χ^2^ = 0.223	0.637
No	2148 (91.87)	1934 (91.75)	214 (93.04)			1921 (91.78)	227 (92.65)		
Yes	190 (8.13)	174 (8.25)	16 (6.96)			172 (8.22)	18 (7.35)		
Television time in a week (min), M(Q_1_,Q_3_)	180.00 (120.00,240.00)	180.00 (120.00,240.00)	180.00 (120.00,240.00)	Z = 1.053	0.292	180.00 (120.00,240.00)	180.00 (120.00,240.00)	Z = -0.037	0.971
Area, n(%)				χ^2^ = 0.003	0.955			χ^2^ = 6.844	0.009
Urban	789 (33.75)	711 (33.73)	78 (33.91)			688 (32.87)	101 (41.22)		
Rural	1549 (66.25)	1397 (66.27)	152 (66.09)			1405 (67.13)	144 (58.78)		
Province, n(%)				χ^2^ = 47.000	<0.001			χ^2^ = 58.769	<0.001
Eastern provinces (Beijing, Shanghai, Jiangsu, Shandong, Zhejiang)	452 (19.33)	374 (17.74)	78 (33.91)			372 (17.77)	80 (32.65)		
Central (Henan, Hubei, Hunan)	633 (27.07)	580 (27.51)	53 (23.04)			574 (27.42)	59 (24.08)		
Western (Guizhou, Guangxi, Chongqing, Shaanxi, Yunnan)	699 (29.90)	662 (31.40)	37 (16.09)			668 (31.92)	31 (12.65)		
Northeastern (Heilongjiang, Liaoning)	554 (23.70)	492 (23.34)	62 (26.96)			479 (22.89)	75 (30.61)		
Hip circumference (cm), Mean±SD	84.23 ± 9.69	83.13 ± 8.84	94.31 ± 11.23	t = -14.61	< .001	82.85 ± 8.62	96.03 ± 10.28	t = -19.29	< .001
Mother’s education level, n(%)				χ^2^ = 24.723	< .001			χ^2^ = 36.420	< .001
None	319 (13.64)	301 (14.28)	18 (7.83)			301 (14.38)	18 (7.35)		
Graduate from primary school	503 (21.51)	460 (21.82)	43 (18.70)			453 (21.64)	50 (20.41)		
Lower middle school degree	958 (40.98)	867 (41.13)	91 (39.57)			864 (41.28)	94 (38.37)		
Upper middle school degree	301 (12.87)	266 (12.62)	35 (15.22)			269 (12.85)	32 (13.06)		
Technical or vocational degree	130 (5.56)	112 (5.31)	18 (7.83)			109 (5.21)	21 (8.57)		
University or college degree or higher	127 (5.43)	102 (4.84)	25 (10.87)			97 (4.63)	30 (12.24)		

BMI, body mass index; WC, waist circumference; SBP, systolic blood pressure; DBP, diastolic blood pressure.

### Association between dietary knowledge and overweight and obesity in children and adolescents

To investigate the relation between dietary knowledge and overweight and obesity in children and adolescents defined by BMI and WC separately, multivariate regression analysis was performed. After correcting for age, gender, and mother’s education level in model 2, dietary knowledge of children and adolescents was significantly associated with overweight or obesity defined by BMI (OR = 1.66, 95%CI = 1.22–2.26, *P* = 0.001), and obesity (OR = 1.49, 95%CI = 1.11–2.00, *P* = 0.008) defined by WC among children and adolescents. Similarly, dietary knowledge of mothers was significantly associated with overweight or obesity defined by BMI (OR = 1.39, 95%CI = 1.02–1.88, *P* = 0.035), and obesity (OR = 1.48, 95%CI = 1.10–1.99, *P* = 0.01) defined by WC in children and adolescents. Then following covariate adjustment in model 3, children and adolescents with low dietary knowledge was demonstrated to have significantly higher risks of BMI-defined overweight or obesity (OR = 1.66, 95%CI = 1.21–2.28, *P* = 0.002), and WC-defined obesity (OR = 1.52, 95%CI = 1.12–2.06, *P* = 0.007) than those with high dietary knowledge. Compared with high dietary knowledge in mothers, low dietary knowledge was associated with significantly elevated risks of BMI-defined overweight or obesity (OR = 1.48, 95%CI = 1.08–2.02, *P* = 0.014), and WC-defined obesity (OR = 1.59, 95%CI = 1.18–2.16, *P* = 0.003) (Table 2).

**Table 2:** Association between dietary knowledge and overweight and obesity in children and adolescents.

	Model 1		Model 2		Model 3	
	OR (95% CI)	*P*	OR (95% CI)	*P*	OR (95% CI)	*P*
Children and adolescents						
BMI	1.82 (1.34–2.65)	<0.0001	1.66 (1.22–2.26)	0.001	1.66 (1.21–2.28)	0.002
WC	1.63 (1.22–2.18)	0.001	1.49 (1.11–2.00)	0.008	1.52 (1.12–2.06)	0.007
Mothers						
BMI	1.55 (1.15–2.09)	0.004	1.39 (1.02–1.88)	0.035	1.48 (1.08–2.02)	0.014
WC	1.64 (1.23–2.20)	<0.0001	1.48 (1.10–1.99)	0.01	1.59 (1.18–2.16)	0.003

For BMI: Model 1, univariate model; Model 2, adjusted for age, gender, and mother’s education level. Model 3, adjusted for age, gender, mother’s education level, geographic location (area and province), systolic blood pressure, and diastolic blood pressure.

For WC: Model 1, univariate model; Model 2, adjusted for age, gender, and mother’s education level. Model 3, adjusted for age, gender, mother’s education level, geographic location (area and province), systolic blood pressure, diastolic blood pressure, and television time in a week.

BMI, body mass index; WC, waist circumference; OR, odds ratios; CI, confidence interval.

### Association between dietary knowledge and overweight and obesity in children and adolescents defined by both BMI and WC

Further, we explored the association between dietary knowledge and overweight and obesity in children and adolescents defined by BMI and WC together. As presented in Table 3, after adjustment for age, gender, mother’s education level, geographic location, SBP, DBP, and television time in a week in model 3, low dietary knowledge of children and adolescents was associated with a significantly higher risk of overweight or obesity defined by BMI or obesity defined by WC in children and adolescents than those with high dietary knowledge (OR = 1.64, 95%CI = 1.26–2.14, *P* < 0.0001). Dietary knowledge of children and adolescents was inversely associated with odds of overweight and obesity in children and adolescents who was overweight or obese defined by BMI and obese defined by WC (OR = 1.60, 95%CI = 1.06–2.43, *P* = 0.025). Significantly increased odds of BMI-defined overweight or obesity and WC-defined non-obesity in children and adolescents were found to be related to low dietary knowledge versus high dietary knowledge of children and adolescents (OR = 1.72, 95%CI = 1.08–2.74, *P* = 0.023), while there was no association of BMI-defined non-overweight and non-obesity and WC-defined obesity with dietary knowledge among children and adolescents (OR = 1.35, 95%CI = 0.89–2.04, *P* = 0.161). Additionally, low dietary knowledge of mothers was significantly associated with BMI-defined overweight or obesity or WC-defined obesity in children and adolescents (OR = 1.58, 95%CI = 1.22–2.06, *P* < 0.0001). A significantly greater risk of BMI-defined overweight or obesity and WC-defined obesity in children and adolescents was in relation to low dietary knowledge rather than high dietary knowledge in mothers (OR = 1.51, 95%CI = 1.01–2.28, *P* = 0.047). No association was identified between dietary knowledge of mothers and BMI-defined overweight or obesity and WC-defined non-obesity among children and adolescents (OR = 1.39, 95%CI = 0.89–2.17, *P* = 0.155), while low dietary knowledge of mothers was associated with increased odds of BMI-defined non-overweight and non-obesity and WC-defined obesity in children and adolescents (OR = 1.58, 95%CI = 1.03–2.43, *P* = 0.036).

**Table 3:** Association between dietary knowledge and overweight and obesity in children and adolescents defined by both BMI and WC.

	Model 1		Model 2		Model 3	
	OR (95% CI)	*P*	OR (95% CI)	*P*	OR (95% CI)	*P*
Children and adolescents						
WC = 1 or BMI = 1	1.73 (1.35–2.23)	<0.001	1.59 (1.23–2.05)	<0.001	1.64 (1.26–2.14)	<0.0001
WC = 1, BMI = 1	1.76 (1.18–2.61)	0.005	1.57 (1.05–2.35)	0.028	1.60 (1.06–2.43)	0.025
WC = 0, BMI = 1	1.78 (1.13–2.81)	0.013	1.67 (1.06–2.65)	0.029	1.72 (1.08–2.74)	0.023
WC = 1, BMI = 0	1.43 (0.95–2.14)	0.088	1.34 (0.89–2.02)	0.165	1.35 (0.89–2.04)	0.161
Mothers						
WC = 1 or BMI = 1	1.60 (1.26–2.08)	<0.001	1.46 (1.13–1.89)	0.003	1.58 (1.22–2.06)	<0.0001
WC = 1, BMI = 1	1.59 (1.08–2.36)	0.019	1.40 (0.94–2.08)	0.099	1.51 (1.01–2.28)	0.047
WC = 0, BMI = 1	1.43 (0.92–2.21)	0.11	1.32 (0.85–2.05)	0.234	1.39 (0.89–2.17)	0.155
WC = 1, BMI = 0	1.60 (1.06–2.43)	0.027	1.50 (0.98–2.29)	0.06	1.58 (1.03–2.43)	0.036

Model 1, univariate model; Model 2, adjusted for age, gender, and mother’s education level. Model 3, adjusted for age, gender, mother’s education level, geographic location (area and province), systolic blood pressure, diastolic blood pressure, and television time in a week.

For BMI: 0 was defined as non-overweight and non-obesity; 1 was defined as overweight or obesity.

For WC: 0 was defined as non-obesity; 1 was defined as obesity.

BMI, body mass index; WC, waist circumference; OR, odds ratios; CI, confidence interval.

## Discussion

In order to investigate the relationship of dietary knowledge and overweight and obesity in Chinese children and adolescents, this study applied data from the CHNS and utilized BMI and WC to assess overweight and obesity. It was revealed that dietary knowledge of children and adolescents and their mothers was associated with their overweight and obesity. Further, low dietary knowledge of children and adolescents related to an elevated risk of BMI-defined overweight or obesity, and dietary knowledge of mothers was negatively associated with odds of WC-defined obesity.

Studies using anthropometric measurements have found that nutrition knowledge is related to BMI and WC, which are the indicators of obesity and its comorbidities, such as cardiovascular diseases [26, 27]. The important associations between knowledge about diets, BMI and WC of children and adolescents in this study highlight the need to develop independence in children and adolescents and to improve nutritional knowledge among mothers, particularly by providing knowledge that enables healthy food selections and implementation of the disease prevention and health promotion programs. Of note, BMI, a most frequently used simple measure of adiposity, has high specificity but low sensitivity to detect excess adiposity and fails to identify over a quarter of children with excess body fat percentage [20]. BMI measures presumed excess weight given height, rather than actual body fat, and does not give any indication as to the distribution of fat in the body, and central adiposity is more closely associated with health risks than general adiposity [21, 28]. As an alternative simple tool to measure adiposity or obesity, WC has a known relation to the amount of abdominal fat, and is of high value as an indicator of risks for developing cardiovascular diseases [27]. The present study identified the association between dietary knowledge of children and adolescents and BMI-defined overweight or obesity, and the association between dietary knowledge of mothers and WC-defined obesity, indicating that improvements (for example, through targeted education and counseling) in the dietary knowledge level of children and adolescents as well as their mothers may help manage central obesity and general obesity, respectively. To this end, (1) schools can set up courses on diets, so that children and adolescents can have more opportunities to enrich their dietary knowledge, thereby improving or preventing overall adiposity. An assessment system can also be built to achieve a good teaching effect; (2) dietary knowledge can be widely publicized in living communities and social networks for mothers to acquire more knowledge on diets, and mothers can also pay more attention to children’s WC, so as to manage central obesity for their children.

Dietary knowledge was revealed here to correlate with overweight and obesity in children and adolescents. Bonaccio et al. proposed that individuals with nutrition knowledge showed greater compliance with the Mediterranean diet and lower obesity prevalence [29]. Another study found an inverse association between nutrition knowledge and BMI [27]. On the other hand, other studies did not found such a relationship [30, 31]. The inconsistency with our findings may lie in differences in populations and sample sizes. Further studies are needed to verify the association of dietary knowledge with overweight and obesity in children and adolescents.

We assumed that the dietary knowledge level affects diet quality and physical activities, which influence childhood and adolescent overweight and obesity [8, 32]. A positive association between nutrition knowledge and dietary intake in adults was shown by Spronk et al. [33], and this association may also exist among children and adolescents, which may further relate to childhood and adolescent overweight and obesity. A CHNS study illustrated that dietary patterns were associated with obesity in Chinese children and adolescents [34], and knowledge about diets may be linked to dietary patterns among children and adolescents. Children and adolescents with high dietary knowledge could have strong self-discipline, which may be associated with their diets and physical activities, and further with overweight and obesity. Mothers with high dietary knowledge can also enable their children to eat healthy and exercise moderately for weight control. There is a relationship between nutritional knowledge, attitudes, and practices, while nutritional attitudes and practices are more relevant than knowledge. Thus, it is important to develop proper strategies not only to improve individuals’ nutritional knowledge but also to enhance their nutritional practices [35, 36]. Individuals with better healthy eating habits were reported to have higher nutrition knowledge levels [37]. Another study conducted with adults also found that the nutrition knowledge level was related to healthy eating [38]. Tabbakh et al. found that greater nutrition knowledge was associated with higher diet quality, and this association was partially mediated by attitudes toward healthy eating [39]. Nonetheless, it is important to note that nutrition knowledge is more effective in combination with behavioral and motivational strategies when individuals are ready to change their nutritional habits [40], which may be conducive for weight management among children and adolescents.

This study first assessed the association of dietary knowledge of children and adolescents and their mothers with childhood and adolescent overweight and obesity, using the nationally representative CHNS data. K-means clustering was used to cluster the dietary knowledge of children and adolescents, and their mothers into two different groups (low dietary knowledge, high dietary knowledge). Some limitations should be noted when interpreting findings. First, a cause-and-effect relationship could not be determined in this cross-sectional study. Second, stability of weight status was not measured in this study, and the reported weight was assumed to be the long-term result of eating self-regulation. Third, data on dietary knowledge were self-reported, which may over- or under-estimate actual weight status. Fourth, variables such as physical activity were not included in the analysis due to limited information from the CHNS.

## Conclusion

Dietary knowledge of children and adolescents and their mothers was associated with childhood and adolescent overweight and obesity. Further, low dietary knowledge of children and adolescents related to an elevated risk of BMI-defined overweight or obesity, and dietary knowledge of mothers was negatively associated with odds of WC-defined obesity. Additional studies are required to confirm our findings and to clarify the causal relationship between dietary knowledge and overweight and obesity among children and adolescents.

## Supporting information

S1 Checklist (DOCX)
